# A case study of global health at the university: implications for research and action

**DOI:** 10.3402/gha.v7.24526

**Published:** 2014-08-27

**Authors:** Andrew D. Pinto, Donald C. Cole, Aleida ter Kuile, Lisa Forman, Katherine Rouleau, Jane Philpott, Barry Pakes, Suzanne Jackson, Carles Muntaner

**Affiliations:** 1 Department of Family and Community Medicine, St. Michael's Hospital, Toronto, Ontario, Canada; 2 Centre for Research on Inner City Health, Keenan Research Centre, Li Ka Shing Knowledge Institute, St. Michael's Hospital, Toronto, Ontario, Canada; 3 Department of Family and Community Medicine, Faculty of Medicine, University of Toronto, Toronto, Ontario, Canada; 4 Dalla Lana School of Public Health, University of Toronto, Toronto, Ontario, Canada; 5 Department of Family and Community Medicine, Credit Valley Hospital, Mississauga, Ontario, Canada; 6 Comparative Program on Health and Society, Munk School of Global Affairs, University of Toronto, Toronto, Ontario, Canada; 7 Department of Family Medicine, Markham Stouffville Hospital, Markham, Ontario, Canada; 8 Global Health Education Initiative, University of Toronto, Toronto, Ontario, Canada; 9 Bloomberg Faculty of Nursing, University of Toronto, Toronto, Ontario, Canada

**Keywords:** global health, international health, academia, university, collaboration

## Abstract

**Background:**

Global health is increasingly a major focus of institutions in high-income countries. However, little work has been done to date to study the inner workings of global health at the university level. Academics may have competing objectives, with few mechanisms to coordinate efforts and pool resources.

**Objective:**

To conduct a case study of global health at Canada's largest health sciences university and to examine how its internal organization influences research and action.

**Design:**

We drew on existing inventories, annual reports, and websites to create an institutional map, identifying centers and departments using the terms ‘global health’ or ‘international health’ to describe their activities. We compiled a list of academics who self-identified as working in global or international health. We purposively sampled persons in leadership positions as key informants. One investigator carried out confidential, semi-structured interviews with 20 key informants. Interview notes were returned to participants for verification and then analyzed thematically by pairs of coders. Synthesis was conducted jointly.

**Results:**

More than 100 academics were identified as working in global health, situated in numerous institutions, centers, and departments. Global health academics interviewed shared a common sense of what global health means and the values that underpin such work. Most academics interviewed expressed frustration at the existing fragmentation and the lack of strategic direction, financial support, and recognition from the university. This hampered collaborative work and projects to tackle global health problems.

**Conclusions:**

The University of Toronto is not exceptional in facing such challenges, and our findings align with existing literature that describes factors that inhibit collaboration in global health work at universities. Global health academics based at universities may work in institutional siloes and this limits both internal and external collaboration. A number of solutions to address these challenges are proposed.

Globalization has accelerated universities’ reach in recent decades. National organizations of educational institutions have taken a role in promoting international partnership programs (1), primarily bilateral and consortia relationships (2). Activities have included the recruitment of students from other countries (3), sending students overseas (4), and greater mobility and joint production of graduate students (5–7). Casting universities’ response to globalization as ‘internationalization’, Knight (5) has noted that ‘internationalization brings new opportunities, new benefits, new risks, and new challenges’.

One arena of internationalization is ‘global health’, a term that has gained ascendancy in high-income countries (8–10). The first occurrence of a university using the term ‘global health’ in the name of a center or institution was in 1999, and by 2009, at least 41 universities in the United States and Canada had established pan-university global health institutes or centers and 11 more had established global health programs in existing departments or divisions (11). Figure 1 sets out various approaches, based on a scan of organizational forms. As Merson and Page note, ‘university-wide centers have expanded the disciplinary framework for global health beyond the health professions to include business, engineering, public policy, divinity, law, and the disciplines of social science’ (11, p. 2). The expansion of this field relates to changes beyond the walls of the academy, including the globalized nature of health, the rapid dissemination of news, the increased inter-connectedness between people, and the framing of global health as a foreign policy objective (12–14). Manifestations of university global health activity include substantial student, faculty, and university presence at global health conferences; the development of research networks; and new coalitions of universities (15–19). There has been a sustained demand for global health education for a number of years in countries around the world (20–23). Students within the health professions, public health, anthropology, social sciences, law, and political science are increasingly undertaking part of their training abroad (24–26). Aside from academics, a complex mix of actors shape the global health agenda, including donors and funding bodies, non-governmental organizations (NGOs), advocacy groups, health professional organizations, private corporations, and governments at all levels.

**Fig. 1:**
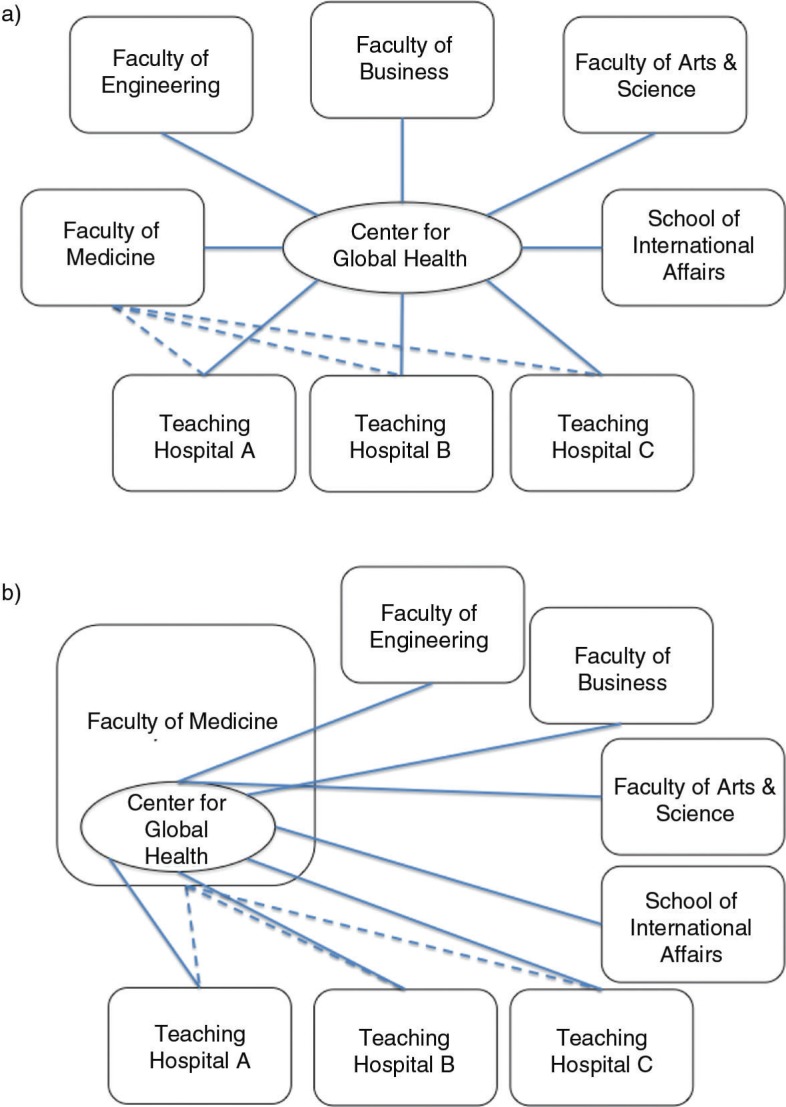
Organization of global health at select universities. a) Emory University, Harvard University, Johns Hopkins University, Yale University, University College London. b) Boston University, Brown University, Columbia University, George Washington University.

Many universities see global health as part of their role as institutions in a global community which have a social accountability or social responsibility mandate (27, 28). They can do so through scholarly work (knowledge generation and its dissemination), education, and decision-maker influence (29, 30). Universities may manage substantial funds and grants, bring together partners from different sectors, and deliver services in high- and low-income countries, often via third-party NGOs. They are also key actors in the economy, for example, through partnering with private enterprise (31). Universities may also innovate better ways to address global health problems and then act to implement their findings (32).

In early 2011, we came together as a group of global health academics and trainees, interested in ‘the state of global health’. In particular, we sought to explore ongoing challenges in managing global health at a large, research intensive university. We hypothesized that how global health is organized influences the research and action of academics, their ability and interest in collaboration internally, and the formation of external partnerships.

## Methods

To conduct this case study we assembled a research team comprised of six faculty members from different disciplines (family medicine, public health, health promotion, nursing, international law/human rights, and ethics) at different stages of their careers, one medical student, and one resident, all based at the University of Toronto. We represent an ‘insider view’ on this issue, bringing our collective years of experience within the institution to bear on the design of the study and the analysis of the results. We obtained ethics approval from the University of Toronto Health Sciences Research Ethics Board.

We assembled a history of global health at the university based on official documents and the knowledge of research team members. Using existing inventories and annual reports, and broad searches of the main website of the University of Toronto, we identified current centers, departments, and hospitals affiliated with the university as potential sites of global health activity in the first half of 2011. A list of faculty was compiled who met the following criteria: 1) affiliation with a center, department, or hospital that was specifically focused on global health or international health, or 2) usage of the terms ‘global health’ or ‘international health’ to describe their work. We recognized that given the myriad forms and decentralized nature of global health activities, we were likely unable to identify every single person engaged in global health at the university. Nevertheless, we listed more than 100 academics engaged in global health situated across the university, often with multiple affiliations (Supplementary file).

From this list, we purposively sampled those in centers, departments, and hospitals from a range of fields to conduct key informant interviews. We sought faculty who were ‘on the ground’ but were also providing academic leadership in global health. One investigator (AP) carried out confidential, semi-structured interviews with each key informant during the summer and fall of 2011. The interview guide included questions about their views of global health; the institution they worked for and its relationship to the university; their experiences in global health activities, including collaboration with other divisions or colleagues; and their thoughts on what helps move global health forward at the university. Because the study was unfunded, the interviews were not audio-recorded and then transcribed, but rather detailed notes of responses were taken during the interview and returned to participants for verification. A pair of coders (AP, AtK) analyzed responses to questions thematically and compiled key themes. Synthesis involved discussion among the research team through in-person meetings and over email.

## Results

### Global health at the University of Toronto (1990–2011)

The University of Toronto is currently Canada's largest academic center, with more than 66,000 undergraduate students, 15,000 graduate students, and 11,000 faculty (33). Historically, clinical research leaders made important contributions to health (34) and held important roles in international health. The School of Hygiene, now the Dalla Lana School of Public Health, was one of a handful established by the Rockefeller Foundation in the 1920s (35). In 2001, the Dean established a Center for International Health, providing core funding to a full-time director, a group of part-time faculty leaders across affiliated teaching hospitals and some departments, and administrative staff. The center became an information-coordinating body for global health by cataloguing activities occurring in different faculties, departments, and hospitals; sharing information online and in annual reports; providing part-time support to faculty members; holding annual global health research days; and leading university-wide initiatives such as World AIDS Day and the University of Toronto HIV/AIDS initiative in Sub-Saharan Africa. The center supported other institutions at affiliated hospitals to apply for grants and external funding for research and training. Resources provided to the Center for International Health at the University of Toronto were much less in comparison to similar institutions in the US (36). As center core funds diminished, global health activities proliferated, most based within teaching hospitals (37). Within the Faculty of Medicine, at least five departments developed one or more programs related to global health. At least four separate global health-related educational programs existed for students at the university, and academics with an interest in this area were found in almost every faculty. Each of these in turn has multiple connections with low- and middle-income country (LMIC) partner universities, hospitals, and NGOs (38). In this context, global health academics at the University of Toronto worked to develop multiple research agendas and launch mostly separate interventions.

### Key informant interviews

To explore how the organization of global health at the university influenced academics’ research and action, we conducted key informant interviews. Among the 28 academics invited to an interview, 25 accepted the invitation. Twenty interviews could be arranged during our study period, of which 19 were in person and one was via telephone. Of the 20 key informants interviewed, 13 (65%) were female. Ten were assistant professors, four were associate professors, four were professors, and two were emeritus professors. Nine cited a teaching hospital as their primary affiliation, four were located within the university's school of public health, and two were with NGOs. One each was with the faculty of nursing, the school of rehabilitation sciences, and the school of international relations. One key informant was predominantly a university administrator.

When asked to define ‘global health’, a fair degree of consensus was apparent among academics, although at least three expressed skepticism about the usefulness of the term ‘global health’, reflecting continued questioning about a shared definition (10, 39, 40). The concept of achieving health equity underpinned most responses. Eight academics explicitly defined the overall objective of work within the field as achieving equity in health outcomes between different populations. Most emphasized that this included vulnerable or marginalized groups both within Canada and within LMIC. Furthermore, several academics included the concept of the social determinants of health in their definition, as well as the idea of multiple, interacting systems influencing health. For example, one stated, ‘it relates to interactions between political jurisdictions and players like NGOs, corporations that are affecting the health of people all over the world’.

Regarding values that should underpin global health work, half of those interviewed cited ‘equity’ as central, and ‘human rights’ and ‘justice’ were each mentioned by five academics. Solidarity, mutual respect, reciprocity, and non-maleficence were common themes in the responses as well. Similar concepts were reported when academics were asked about what they emphasize in their work, with many additionally citing the importance of sustainability.

More than half of those interviewed reported that they saw themselves as collaborating with others at the university, or as members of a team within the university. The remainder felt that they were working more or less independently from others, often emphasizing that they felt isolated from others’ work. Many in both groups felt that their work contributed to the university's mandate on global health, although this was predominantly around research. Those whose work predominantly involved developing education initiatives felt left out. Many assessed the university's leadership to be increasingly supportive of global health, at least in principle, as reflected in recent strategic plans.

All 20 academics expressed a negative assessment of how global health was currently organized at the University of Toronto. They perceived that their work existed in silos or in parallel to others with a lack of support from the university. Descriptors used included ‘disjointed’, ‘incoherent’, ‘fragmented’, ‘chaos’, and ‘anarchic’. ‘We lack vision with lots of small initiatives, but not an overarching system, structure, or principles to bring us together and create synergy’. The primary method of organizing global health efforts was noted to be around key individuals rather than specific institutional structures. Given the challenges in collaboration, several academics interviewed also noted ‘glimmers of hope’. Most felt that there were many people working hard but not in collaboration with one another. An exemplar of this sentiment was, ‘There are lots of people doing good things. There hasn't been a cohesive umbrella that would help catalyze and synthesize. There are people doing their own thing, and there is nothing wrong with that. It could be more efficient to use resources to have a greater impact’.

Barriers to collaboration with colleagues within the university that were highlighted by the interviewed academics included the absence of an overall strategic plan from the university around global health (at the time), a lack of time and opportunity to connect with colleagues, and limited incentives for collaboration. The large institutional size of the University of Toronto also made it difficult to connect with others. Several academics noted that fragmentation may not always be negative, in that it may facilitate multiple approaches to a problems based on different approaches to working in global health. Although some academics could not identify any existing factors that facilitated connecting with others, many identified the role of individuals acting as facilitators as essential to forming linkages; these were referred to as ‘champions’. Also cited as important were regular meetings or forums to meet colleagues and building on personal networks. Many cited the enthusiasm of others and their willingness to share time and resources as important facilitating factors.

Those interviewed highlighted that international collaboration occurred primarily through personal and individual effort, rather than through an institutional process. Most academics that worked with colleagues outside of Canada felt that the university had not played a substantial role in establishing these relationships. Some even went so far as to indicate that the university policies and processes might have had a negative impact on such relationships. Some noted concerns around the institution's adverse influence on partnership development, including unduly onerous bureaucracy, a lack of resources, and limited recognition for their efforts.

No single solution was identified to resolve concerns about the organization of global health at the university. Broad sets of ideas proposed included developing mechanisms or structures to help people connect and share ideas and resources, such as regular meetings that would improve dialogue and communication across the university around global health. Many suggested establishing a better sense of what is happening around global health currently. For example, helping academics know about existing external partnerships. Several academics recommended that the university propose incentives to promote internal and external collaboration. Most academics recommended that the university prioritize better coherence and develop a shared strategic plan around global health. Similar to the discussion of individual supports, many academics identified funding and support for faculty and students as important priorities. Some articulated a need for systems thinking that brings together individual academics into teams that can address the complexity of global health problems (41). Leadership that can balance the needs of faculty and funders, university administrators, the academic community, philanthropists, and advocates was seen as needed, as well as the mobilization of new resources (42).

Many academics felt a strong, university-wide center for global health would be helpful. Examples of successful centers provided included ones at Johns Hopkins, Harvard, London School of Hygiene and Tropical Medicine, and specific institutions such as the Earth Institute (Columbia University), and François-Xavier Bagnoud Center for Health and Human Rights (Harvard University). It was proposed that such a center could provide supports to academics and be a place to share ideas. It could also help in developing and driving a shared vision and strategic plan, working with stakeholders to clarify areas for collaboration, resource development and adding value, as has been developed in collaborative research centers (43).

However, several academics were also wary of too much control over individual academic agendas. A tension was evident in the responses given and the misgivings of faculty towards such a center would need to be carefully addressed through a strategic planning process. Such tensions are apparent in other academic planning processes in higher education institutions, which value the integrity of academic inquiry, led by individuals, at the same time wanting to benefit from the stimulation of cross-disciplinary initiatives and resource mobilization that can come through working together.

## Discussion

We have presented a case study of how global health activities have been organized at the University of Toronto. Through key informant interviews, we established that there is consensus among academics from a variety of disciplines and centers that fragmentation and siloed efforts are a major concern. Not only does this limit joint efforts through internal collaboration, it hampers the establishment of external partnerships. The lack of a central vision has potentially hampered the mobilization of the substantial resources of the university toward taking large-scale action in global health. A well-resourced, university-wide center was identified as one potential solution by our participants.

Subsequent to our study, a strategic planning process did get underway in the Faculty of Medicine (38). Our initial findings were used in the development of a ‘roadmap’ for global health in the Faculty of Medicine. Furthermore, a new Institute for Global Health and Equity and Innovation has been proposed, based at the Dalla Lana School of Public Health, but with cross-university participation. A global health summit to engage all academics and institutions across the university in the development of such an Institute is schedule for November 2014. It remains unclear what the Institute will look like, but much can be learned from the experience of others. At Emory University (Atlanta, Georgia, USA), after deliberations involving faculty, staff, students, and alumni on the role of the university in global health, a Global Health Institute was developed. Substantial start-up funds were obtained and a clear vision and strategic plan was developed with explicit performance metrics. An internal advisory committee specifically works to foster cross-unit cooperation and resolve barriers to collaboration (28). At Johns Hopkins University (Baltimore, MD, USA) the center for global health was developed as a hub that ‘interdigitates’ with medicine, public health, and nursing. The center has explicitly laid out objectives that include a multidisciplinary approach to solving global health problems (44). At Vanderbilt University (Nashville, Tennessee, USA), the Institute for Global Health took a ‘center-without-walls’ approach to nurture non-competitive partnerships among and within departments and schools. Part of the role of the Institute is to maintain an ongoing repository of global health activities across the university (45). At the University of California, San Francisco (San Francisco, CA, USA), global health sciences operates across dentistry, medicine, nursing, and pharmacy, supported by existing centers and institutes whose directors serve on its executive committee. A database of ongoing projects, faculty experience, and interests is maintained, and the focus is on demonstrating the value-add of participation rather than increasing competition (46). Finally, the University of Virginia (Charlottesville, VA, USA) established a center for global health building on past experience with international health. The center ensures that there are dedicated personnel to provide leadership and coordinate communication and collaboration among faculty, administrators, and students across departments and schools (47).

We note several limitations to our study. The findings may not be generalizable to other institutions that differ in size, composition history, and context. However, we feel that the views of academics captured here may be similar to others working in global health at other universities. We conducted only 20 interviews, representing a subset of all disciplines involved in global health, but we found a great deal of consistency in responses, and did not feel that further interviews would reveal new themes. Finally, the organization of global health at the University of Toronto is continually changing, and this study presents only a cross-sectional view. Longitudinal research that tracked the evolution of collaboration in this area would be valuable to academics and administrators. Bibliometric analysis could provide greater insight into existing and potential networks of academics and how this changes over time, including how academics situated in medical and non-medical institutions do or do not collaborate (48, 49). Network analysis methods could assist in mapping out collaboration and understanding where information is and is not shared and where improved collaboration could happen (50). The different perspectives and activities that drive ‘global health brands’ at universities could be explored. Finally, organizational researchers could examine the impact of interventions (e.g. strategic planning, small grants, and networking events) on global health collaboration within higher education institutions.

We believe the findings of our exploratory study are particularly relevant to global health leaders developing capacity at their institutions, and trainees who hope to contribute to the field as academics in the future. The University of Toronto is certainly not exceptional in facing such challenges (11, 36, 51). Our findings align with existing literature that describes four key factors that inhibit collaboration in global health work at universities. *First*, institutional cultures may favor discipline-specific funding, where rewards accrue to individuals rather than teams, and foster competition between centers, schools, and departments (29, 52). *Second*, collaboration is complicated further by the lack of a standard definition of global health to cohere efforts (9). Colleagues must be convinced of the validity and sustainability of global health as an academic field (11). As with many new fields, global health has developed organically and often disparately. Even within a single institution, it may be difficult to decide who identifies with the field and in what way. Simply knowing who is working on what and where (geographically) and with whom (organizationally) across different centers or departments can be helpful (51). *Third*, initiatives within a single institution often have different and competing objectives. They may emphasize research, education, or service more than other areas. They may have different views on the role of equity and take different approaches in their relationships with partners (40). Often, no institutional mechanism exists for elucidating – let alone addressing – potential conflicts. *Fourth*, actors must often sacrifice time and energy to coordinate their activities. Such coordination is rarely supported centrally by the institution and may take academics away from their primary activities with partners. These obstacles result in a lack of a sense of ‘community’ among global health academics; a sense of fragmentation for all stakeholders; and inefficiencies in service, research, and education (28).
